# Nursing Students’ Perceptions of Lecturers’ Caring Presence During Online Learning in a Public Nursing College in South Africa

**DOI:** 10.1155/nrp/3235288

**Published:** 2026-09-04

**Authors:** Thembile Precious Ndlovu, Charlene Downing

**Affiliations:** ^1^ Department of Nursing, Faculty of Health Sciences, University of Johannesburg (UJ), Johannesburg, South Africa; ^2^ Department of Nursing, Faculty of Health Sciences, University of Johannesburg (UJ), 55 Beit Street Doornfontein, Johannesburg, South Africa

**Keywords:** caring presence, nursing education, nursing students, online learning, qualitative research

## Abstract

**Background:**

The Diploma in Nursing programme (Regulation R.171) in South Africa has traditionally been delivered through face‐to‐face classroom teaching combined with supervised clinical practice. However, the COVID‐19 pandemic in 2020 necessitated a rapid transition to online learning to ensure continuity of nursing education while minimising the spread of the virus. As caring is a fundamental value underpinning the nursing profession, it was essential that this value be maintained within virtual learning environments. In this study, caring presence is conceptualised as both relational and structural, encompassing lecturers’ communication, emotional support, accessibility, institutional responsiveness and digital equity.

**Aim:**

This study explored nursing students’ perceptions of lecturers’ caring presence during online learning at a public nursing college in Gauteng Province, South Africa, and developed recommendations to inform the design and implementation of future online and blended learning environments.

**Design and Methods:**

A qualitative, contextual research design guided by the Appreciative Inquiry approach was employed to explore nursing students’ experiences of lecturers’ caring presence during online learning. Data were collected through focus group interviews with third‐year nursing students and analysed using thematic analysis.

**Participants and Setting:**

Twenty‐three third‐year nursing students enrolled in the Diploma in Nursing programme participated in four focus group interviews conducted across two nursing college campuses in South Africa.

**Results:**

Four overarching themes emerged from the data, reflecting students’ perceptions of lecturers’ caring presence during online learning. Participants highlighted the importance of effective communication, emotional support, lecturer accessibility, institutional responsiveness and equitable access to digital resources in fostering a caring online learning environment. Students recommended integrating caring values into the college’s mission and philosophy, strengthening the development of professional attributes such as caring, compassion, resilience and empathy through blended learning approaches, incorporating digital literacy into the nursing curriculum, providing continuous professional development for lecturers in the use of learning management systems and ensuring accessible technical support for all nursing students.

**Conclusion:**

The findings demonstrate that caring presence remains a critical component of quality nursing education, regardless of the mode of delivery. Strengthening caring relationships, institutional support and digital preparedness can enhance students’ learning experiences in online environments. The recommendations may inform curriculum development, educational policy and future research aimed at promoting caring‐centred online nursing education.

## 1. Background

The Coronavirus Disease 2019 (COVID‐19) pandemic disrupted higher education globally, compelling universities and nursing education institutions to suspend face‐to‐face teaching and rapidly transition to online learning to ensure continuity of education while limiting virus transmission [1, 2]. This unprecedented shift presented significant challenges for nursing education, where theoretical instruction and clinical learning are closely integrated, and where caring relationships between lecturers and students form an essential component of professional development [3, 4]. Although many higher education institutions successfully adopted online teaching technologies during the pandemic, the transition exposed substantial disparities in institutional readiness, technological infrastructure and digital competence. Institutions with established online learning systems adapted more effectively than those with limited digital capacity [5, 6]. Consequently, nursing students experienced increased academic stress, social isolation, interruptions to clinical learning and reduced opportunities for meaningful interaction with lecturers and peers [7–9].

Countries such as Singapore, which resemble most British Commonwealth Countries with their rapidly changing healthcare landscape, nursing services and nursing education, facilitated a swift change [10]. However, in Nepal, the shift to online learning was challenging as it was generally a new experience for the country, South Africa, as a developing country, faced similar challenges [11–13]. In South Africa, the transition to online nursing education was particularly challenging because many institutions relied predominantly on traditional face‐to‐face teaching before the pandemic [14, 15]. Existing inequalities in access to technology, internet connectivity and digital literacy further widened educational disparities among nursing students [16]. Although national policies have increasingly promoted the integration of information and communication technologies into nursing education, implementation remains inconsistent and many nurse educators continue to encounter challenges in delivering effective online learning [17, 18]. Adopting Information and Communications Technology (ICT) into the nursing curricula will be beneficial as it will guide nurses in future evidence‐based practice that includes a digital‐based model of care [19].

The South African Department of Health developed ICT policies for Nursing Education Institutions (NEIs) to support the nursing curriculum and improve technology integration in the facilitation of learning [20]. The Strategic Plan for Nurse Education, Training and Practice (2012/2013‐2016/2017) was updated to the National Strategic Direction for Nursing and Midwifery Education and Practice (2020/21‐2025/26) in South Africa. It calls for the upgraded use of ICT in nursing and midwifery care in the country [18]. However, lecturers’ reluctance to use these new methods is ongoing [17], even though the COVID‐19 pandemic made it mandatory to use online learning [21].

Nursing students were moved to online platforms during the COVID‐19 pandemic to continue their nursing education at nursing colleges. However, Aboagye [22] discovered that the physical separation of nursing students, social distancing and the new expectations of the online learning system influenced nursing students’ psychological well‐being. In support, Bejster et al. [23] and Khagi et al. [24] reported some challenges with the move to online learning, such as switching courses to online overnight, the inability to address nursing students’ needs outside the traditional classroom, the unavailability of resources and the skills shortages. Li et al. [21] and Mutongoza [25] also discovered that the current nursing curriculum is not designed for online learning, and much still needs to be done to overcome the resultant barriers. Ultimately, lecturers need to design online classroom environments to support nursing students [26, 27]. As online learning gains velocity, it is important to develop an approach that will nurture nursing students and create a virtual community that promotes students’ learning [28, 29].

Caring is recognised as a core value of nursing and underpins both professional practice and nursing education [30]. Within educational settings, caring extends beyond the transmission of knowledge to encompass meaningful interpersonal relationships that foster trust, empathy, emotional support and professional growth [31, 32]. As nursing education increasingly incorporates online and blended learning, maintaining these caring relationships has become both more challenging and more important.

Caring in nursing education manifests practices that acknowledge the various complexities and insights among nursing students that are taught, fostering an attitude that embodies wholeness [31, 32]. Caring presence in online learning involves more than frequent communication between lecturers and students [32]. It encompasses relational dimensions, including empathy, accessibility, respect, encouragement and emotional support, together with structural dimensions such as institutional responsiveness, equitable access to digital resources and the creation of inclusive learning environments. These elements collectively influence students’ sense of belonging, engagement and academic success in virtual learning environments [5, 26].

The caring pedagogy in nursing education nurtures the cogeneration of knowledge, assists in embracing the whole nursing student and acknowledges their learning needs [31, 33].

The literature is available that focuses on online learning during the COVID‐19 pandemic, its benefits and the challenges experienced by both lecturers and nursing students [34, 35]. A study by Bradley et al. [33] focused on caring and compassion in an online nursing environment at the Sacred Heart University College of Nursing in Washington, USA. Another international study at the University of South Carolina in the USA by Jones et al. [34] examined nursing students’ perceptions of faculty caring in online nursing education. Although international studies have examined faculty caring and online nursing education, most evidence originates from high‐income countries such as the United States and Canada [36]. Little is known about how nursing students in South African nursing colleges perceive lecturers’ caring presence during online learning, particularly within educational contexts characterised by resource constraints and digital inequities. Understanding these perceptions is essential for developing contextually relevant teaching strategies that support both academic success and the caring values central to nursing education. Therefore, this study explored nursing students’ perceptions of lecturers’ caring presence during online learning in Gauteng nursing colleges and developed recommendations to inform the design of future online and blended learning environments.

## 2. Methods

### 2.1. Research Design

This study employed a qualitative, contextual research design to explore nursing students’ perceptions of lecturers’ caring presence during online learning. Appreciative Inquiry (AI) was used to guide the focus group interviews and facilitate data collection by encouraging participants to reflect on positive experiences and collaboratively identify strategies for strengthening caring presence in future online learning environments. A qualitative design was considered appropriate because it enabled an in‐depth exploration of students’ experiences, perceptions and meanings associated with the phenomenon under investigation [37, 38]. Qualitative research is particularly suited to addressing questions that seek to understand how and why individuals experience particular phenomena while providing rich descriptions of their lived experiences [39, 40]. The study was conducted within a contextual research design, allowing the phenomenon to be explored within the participants’ natural educational environment [41]. Contextual research seeks to understand experiences within the settings in which they naturally occur and acknowledges the influence of the social and organisational context on participants’ perceptions [42, 43]. Accordingly, focus group interviews were conducted at the participating nursing college campuses, enabling students to discuss their experiences within familiar learning environments.

### 2.2. AI

The AI approach informed the data collection process. AI is a strength‐based participatory approach that focuses on identifying existing strengths and positive experiences while generating ideas for future improvement [44]. Rather than concentrating solely on problems, AI encourages participants to reflect on successful experiences and collaboratively envision strategies for enhancing practice [45]. The study was guided by the 5‐D AI cycle—Definition, Discovery, Dream, Design and Destiny [44]. During the focus group discussions, participants first reflected on positive experiences of lecturers’ caring presence during online learning (Discovery), envisioned ideal caring practices (Dream) and proposed recommendations for strengthening caring presence in future online and blended learning environments (Design and Destiny). The AI approach was particularly suited to exploring caring presence because it encouraged participants to reflect on positive lecturer–student interactions, identify behaviours that fostered a caring online learning environment and collaboratively propose strategies for strengthening caring practices in future online nursing education [46].

Although AI has been criticised for emphasising positive experiences, it does not disregard challenges [47]. Instead, it reframes difficulties as opportunities for learning and improvement [45]. This approach aligned well with the purpose of the study, as it enabled nursing students to acknowledge both the strengths and limitations of their online learning experiences while developing constructive recommendations to enhance lecturers’ caring presence. The AI approach was particularly appropriate for this study because it supports collaborative reflection, promotes positive dialogue and facilitates organisational learning and change within nursing education [45]. Trustworthiness was established by addressing the criteria of credibility, dependability, confirmability and transferability, which are described in a later section of this manuscript [48].

### 2.3. Setting

Qualitative research is mostly conducted in natural settings [49]. Natural settings refer to the participants’ or subject matter’s original context without any manipulations or modifications. It is a familiar setting that offers sufficient space, is comfortable, accessible and has few distractions [49–52]. The study was conducted at two campuses of a public nursing college in Gauteng Province, South Africa. The Gauteng College of Nursing is a public nursing education institution that offers the Diploma in Nursing (Regulation R.171) program, which prepares students for registration as general nurses with the South African Nursing Council (SANC).

During the COVID‐19 pandemic (2020‐2021), teaching and learning were transitioned from traditional face‐to‐face instruction to online learning using digital platforms to ensure continuity of nursing education while complying with national public health regulations. At the time of data collection, the participating third‐year nursing students had completed a substantial portion of their first‐ and second‐year theoretical learning through online modalities, enabling them to reflect on their experiences of lecturers’ caring presence during online learning.

The focus group interviews were conducted in private rooms at the respective nursing college campuses. Conducting the interviews within the participants’ familiar educational environment promoted comfort, accessibility and open discussion while ensuring privacy and minimal interruptions during data collection.

### 2.4. Population and Sampling

The study population comprised third‐year nursing students enrolled in the Diploma in Nursing (Regulation R.171) program at two campuses of a public nursing college in Gauteng Province, South Africa. Third‐year students were purposively selected because they had experienced online learning during their first and second years of study (March 2020 to December 2021), when teaching was delivered virtually in response to the COVID‐19 pandemic. Their experiences positioned them to provide rich and relevant insights into lecturers’ caring presence within the online learning environment. A purposive sampling strategy was employed to recruit participants who had direct experience of the phenomenon under investigation and were able to provide detailed accounts of their experiences [38, 53]. Purposive sampling is widely used in qualitative research to select information‐rich participants whose experiences are relevant to the study’s objectives.

#### 2.4.1. Inclusion Criteria

• Enrolled in the third year of the R.171 Diploma in Nursing program.
• Experienced online learning during the COVID‐19 pandemic.
• Aged 18 years or older.
• Willing to participate and provide informed consent.

#### 2.4.2. Exclusion Criteria

• First‐ and second‐year students.
• Students who had not experienced online learning during the pandemic.
• Students absent during data collection.
• Students who were enrolled in other programs outside the R.171 program.

Following institutional approval, the researcher liaised with the campus heads and research coordinators at the participating campuses to arrange information sessions and identify suitable dates for data collection. Students who met the inclusion criteria received information about the study and were invited to participate voluntarily. Those who agreed provided written informed consent before participation. The final sample comprised 23 third‐year nursing students, including 20 females and three males, all aged 18 years or older. All participants self‐identified as Black South Africans. The sample size was considered adequate because it provided sufficient depth and diversity of experiences to address the study aim, and no substantially new information emerged during the later focus group discussions, indicating data saturation.

### 2.5. Pilot Study

A pilot focus group interview was conducted before commencement of the main study at one of the participating campuses. The purpose of the pilot study was to assess the clarity, relevance and sequencing of the AI interview guide, evaluate the feasibility of the data collection procedures and enable the researcher to develop confidence and competence in facilitating focus group discussions [54]. The researcher initially invited an experienced qualitative researcher with a PhD in nursing to observe the pilot focus group and provide feedback on the interview process. However, the researcher was unavailable at the scheduled time, and the pilot interview was therefore conducted independently by the researcher.

The interview guide consisted of five AI questions aligned with the 5‐D cycle (Definition, Discovery, Dream, Design and Destiny). Following the pilot interview, the researcher reflected on the interview process with the research supervisor. No substantive modifications were required to the wording, sequencing or structure of the interview guide. Instead, the supervisor advised the researcher to allow participants greater opportunity to guide the discussion naturally and to minimise unnecessary interruption during the focus group.

As no changes were made to the interview guide and the pilot participants met the study inclusion criteria, the pilot focus group data were considered relevant to the study objectives and were therefore included in the final data analysis.

### 2.6. Data Collection

Data were collected through four face‐to‐face focus group interviews conducted across two campuses of a public nursing college in Gauteng Province, South Africa. Focus groups were selected because they enabled participants with shared experiences of online learning to discuss their perceptions collectively, build on one another’s responses and generate rich descriptions of lecturers’ caring presence [55, 56].

Each campus hosted two focus group interviews, comprising 4–9 participants per group, resulting in a total of 23 participants. The focus groups were conducted by the researcher between January and April 2024 in private meeting rooms on the respective campuses. Interviews were scheduled after formal teaching sessions to minimise disruption to students’ academic activities. Each focus group lasted approximately 60–120 min.

The AI approach guided the data collection process by encouraging participants to reflect on positive experiences of lecturers caring presence during online learning while collaboratively identifying opportunities to strengthen future online and blended learning practices. The focus group interview guide was developed using the 5‐D AI cycle (Definition, Discovery, Dream, Design and Destiny) [44]. The interview guide was informed by the literature and reviewed by the research supervisor to establish content relevance.

The following open‐ended questions guided the discussions:

• Definition: How would you define lecturers’ caring presence during online learning?
• Discovery: Can you describe your most positive experiences of lecturers’ caring presence during online learning?
• Dream: What caring practices would you like to experience in future online learning environments?
• Design: What strategies should be implemented to promote lecturers’ caring presence in online learning?
• Destiny: How can caring presence be sustained and strengthened in future online learning environments?

Throughout the focus group interviews, the researcher used facilitation techniques including active listening, probing, clarification, reflective responses, summarising and the appropriate use of silence to encourage participants to elaborate on their experiences [38, 57]. As the sole facilitator, the researcher conducted all interviews without an assistant moderator, thereby ensuring consistency in the facilitation of discussions across all four focus groups. With participants’ written consent, all interviews were audio‐recorded and transcribed verbatim. Field notes documenting nonverbal communication, group interactions and contextual observations were recorded immediately after each focus group to complement the interview transcripts during data analysis. Data collection continued until data saturation was achieved, defined as the point at which no substantially new themes or insights emerged from successive focus group discussions [58, 59]. Saturation was reached after the fourth focus group interview, a finding that was subsequently confirmed during independent coding of the data. An independent qualitative researcher experienced in qualitative data analysis coded the transcripts independently. Differences in coding were discussed until consensus was reached. Participant characteristics are presented in Table 1.

**TABLE 1:** Participants’ characteristics.

Characteristic	*n* (%)
Participants	23
Female	20 (87%)
Male	3 (13%)
Age ≥ 18 years	23 (100%)
Black South African	23 (100%)
Third‐year students	23 (100%)
Experienced online learning during COVID‐19	23 (100%)

### 2.7. Data Analysis

Data were analysed using Braun and Clarke’s [60] six‐phase thematic analysis, which provides a systematic approach for identifying, analysing and reporting patterns of meaning within qualitative data. All focus group interviews were audio‐recorded and transcribed verbatim. The researcher repeatedly read the transcripts while reviewing the accompanying field notes to become familiar with the data and gain an overall understanding of participants’ experiences of lecturers’ caring presence during online learning.

The six phases of thematic analysis were followed:

1. Familiarisation with the data: The researcher immersed herself in the data by reading and rereading the transcripts while listening to the audio recordings to ensure accuracy and gain a comprehensive understanding of participants’ experiences.
2. Generating initial codes: Meaningful segments of text relevant to the research question were systematically coded across the dataset. Reflective journal entries and field notes were used to support the coding process and enhance reflexivity.
3. Searching for themes: Related codes were grouped according to conceptual similarity to develop preliminary themes and subthemes that represented recurring patterns across the data.
4. Reviewing themes: Preliminary themes were reviewed against the coded extracts and the complete dataset to ensure internal consistency and clear distinctions between themes. Themes with insufficient supporting data were merged, refined or discarded where appropriate.
5. Defining and naming themes: Each theme was clearly defined and named to accurately reflect its underlying meaning and relationship to nursing students’ perceptions of lecturers’ caring presence during online learning.
6. Producing the report: The final themes were organised into a coherent narrative supported by representative participant quotations to illustrate the findings.

To enhance the trustworthiness of the analysis, the researcher engaged in regular discussions with the research supervisor throughout the analytical process (Amin et al.) [61]. The researcher transcribed all focus group interviews verbatim [62]. An independent coder, who was an academic with a doctoral qualification and extensive experience in qualitative research, independently analysed the interview transcripts. The researcher and the independent coder compared and discussed the coding, categories and themes until consensus was reached, thereby enhancing the credibility and dependability of the findings [63]. Although AI informed the focus group discussions, it was not used as the analytical framework. The participants’ responses generated during the Definition, Discovery, Dream, Design and Destiny phases informed the content of the thematic analysis and contributed to the development of the final themes.

### 2.8. Researcher Reflexivity

The researcher is a nurse educator with experience in nursing education and online teaching. Throughout the study, reflexivity was maintained by keeping a reflective journal to document methodological decisions, personal reflections and potential assumptions that could influence data collection and analysis [64]. Regular discussions with the research supervisor and independent coder further promoted critical reflection and enhanced the trustworthiness of the findings.

### 2.9. Trustworthiness

The trustworthiness of the study was established using the four criteria proposed by Lincoln and Guba [65] credibility, transferability, dependability and confirmability.

### 2.10. Credibility

Credibility refers to confidence in the truthfulness of the findings. It was enhanced through prolonged engagement with participants during the focus group interviews, which lasted between 60 and 120 min and enabled in‐depth exploration of nursing students’ perceptions of lecturers’ caring presence during online learning. The researcher also maintained field notes and a reflective journal throughout the research process to document observations and methodological decisions. Regular peer debriefing with the research supervisor provided opportunities to discuss emerging interpretations and challenge assumptions. In addition, an independent qualitative researcher with a doctoral qualification and extensive qualitative research experience independently coded the transcripts, after which coding and themes were compared and discussed until consensus was reached. Although transcript review by participants (member checking) was planned, it was not feasible because participants were unavailable for follow‐up meetings. Consequently, credibility was strengthened through the combination of prolonged engagement, reflexive journaling, supervisor consultation and independent coding.

### 2.11. Transferability

Transferability was enhanced by providing a rich description of the study context, setting, participants, sampling strategy and data collection procedures. Such detailed contextual information enables readers to determine the applicability of the findings to other nursing education settings.

### 2.12. Dependability

Dependability was promoted by maintaining a detailed audit trail documenting each stage of the research process, including methodological decisions, data collection procedures, transcription, coding and theme development. The independent coding process and regular discussions with the research supervisor further strengthened the consistency and transparency of the analytical process.

### 2.13. Confirmability

Confirmability was established by maintaining an audit trail comprising audio recordings, verbatim transcripts, field notes, reflective journal entries, coding records and theme development documents. The independent coding process and regular consultation with the research supervisor helped ensure that the findings reflected participants’ experiences rather than the researcher’s personal assumptions or biases.

### 2.14. Ethical Considerations

Ethical approval for the study was obtained from the University of Johannesburg Faculty of Health Sciences Research Ethics Committee (REC‐1670‐2022) and the Higher Degrees Committee (HDC‐01‐55‐2022) before commencement of the study. Permission to conduct the research was subsequently granted by the Gauteng Department of Health Provincial Protocol Review Committee (GP202208_072) and the management of the participating campuses of a public nursing college in Gauteng Province, South Africa.

### 2.15. Informed Consent

Eligible participants received written and verbal information explaining the purpose of the study, research procedures, potential risks and benefits and their rights as research participants. Written informed consent was obtained from all participants before data collection commenced [66]. Separate consent was obtained for the audio‐recording of the focus group interviews. Participation was entirely voluntary, and participants were informed of their right to decline participation or withdraw from the study at any stage without academic or personal consequences [67]. Participants also provided consent to be contacted via WhatsApp for scheduling focus group interviews [68]. Permission to distribute participant information sheets electronically and in hard copy was obtained from the relevant campus gatekeepers in accordance with the Protection of Personal Information Act (POPIA) (Act No. 4 of 2013). The researcher had no direct teaching or assessment responsibilities for the participants during the study.

### 2.16. Privacy and Confidentiality

Focus group interviews were conducted in private rooms on the participating campuses to ensure a comfortable, confidential and nonthreatening environment. Although anonymity could not be guaranteed because participants knew one another within the focus groups, confidentiality was emphasised before each discussion [69]. Participants were requested to respect the privacy of fellow group members by not disclosing information shared during the discussions outside the group [70]. Interview transcripts were anonymised using participant codes, and all identifying information was removed before analysis. The researcher transcribed all audio recordings verbatim while maintaining participant confidentiality throughout the transcription process. Audio recordings, transcripts, field notes and consent forms were accessible only to the researcher and an independent qualitative researcher who signed a confidentiality agreement before accessing the data. The focus groups were conducted in English. Where participants expressed themselves in Setswana or Sesotho, these responses were translated into English during transcription while preserving the original meaning and context. Translations were undertaken by the researcher, who was fluent in the languages used during the interviews.

### 2.17. Risks and Benefits

The study posed minimal risk to participants [71]. Although discussions regarding online learning experiences had the potential to evoke emotional discomfort, appropriate support mechanisms were available through the student counselling services if required. No participant experienced significant emotional distress or requested counselling during the study. While participants received no financial incentive for participation [72], they were afforded the opportunity to reflect on their experiences and contribute recommendations to strengthen lecturers’ caring presence in future online and blended nursing education.

### 2.18. Data Management

Electronic data, including audio recordings and interview transcripts, were stored on password‐protected and encrypted devices. Hard‐copy documents, including signed consent forms and field notes, were stored in a locked cabinet accessible only to the researcher. The independent qualitative researcher was granted access to the data only after signing a confidentiality agreement. Data will be securely retained for a minimum of 5 years following completion of the study in accordance with the University of Johannesburg’s research governance requirements, after which they will be securely destroyed or archived in accordance with institutional policy [73].

## 3. Results

The study explored and described nursing students’ perceptions of lecturers’ caring presence during online learning at two campuses of the Gauteng College of Nursing during the COVID‐19 pandemic. Data were analysed using Braun and Clarke’s six‐phase thematic analysis, which generated three themes and nine subthemes. Although the focus group discussions were guided by the AI 5‐D framework, the findings were organised inductively into themes that reflected participants’ experiences of lecturers’ caring presence during online learning. Three predominant themes became apparent.

### 3.1. Theme 1

Participants experienced caring and uncaring moments from their lecturers.

### 3.2. Theme 2

Participants experienced that NEIs’ sudden shift to online learning as the only teaching tool and numerous other factors contributed to a chaotic academic process.

### 3.3. Theme 3

Participants expressed their academic, technological and personal needs to succeed in future technological learning environments.

The three predominant themes are presented in Table 2 below:

**TABLE 2:** Summary of themes and interpretation.

Theme	Subthemes	Interpretation
Theme 1: Perceptions of lecturers’ caring presence during online learning	Caring communication; Emotional support; Responsive teaching	Caring presence was primarily experienced through meaningful lecturer–student relationships and effective communication.
Theme 2: Experiences of the transition to online learning	Institutional preparedness; Positive learning experiences; Technological challenges	Students’ online learning experiences were influenced by both personal resilience and institutional readiness.
Theme 3: Recommendations for strengthening future online learning	Student support; Lecturer development; Institutional leadership	Caring online learning requires coordinated efforts from educators, institutions and policy makers.

### 3.4. Theme 1: Participants Experienced Caring and Uncaring Moments From Their Lecturers

Participants described both positive and negative experiences of lecturers’ caring presence during online learning. Caring presence was reflected in lecturers’ accessibility, responsiveness, willingness to adapt to unfamiliar online teaching methods and efforts to maintain communication despite the challenges of the COVID‐19 pandemic. Conversely, participants perceived a lack of caring when academic support was limited, communication was ineffective and their emotional well‐being received little attention during the transition to online learning.

#### 3.4.1. Subtheme 1.1: Participants Experienced Caring Moments From the Lecturers

Participants described lecturers’ caring presence as being demonstrated through behaviours that promoted accessibility, flexibility and ongoing academic support during online learning. Recording lectures, responding promptly to emails and messages and remaining available for consultation outside normal teaching hours were viewed as expressions of genuine concern for students’ learning and well‐being.

One participant explained:

“So I particularly liked one lecturer because she would go an extra mile of like recording her content via the WhatsApp uhmmmm voice notes, so for me it was so nice because I could literally like listen to what she is saying when I am like busy cleaning, understand the content better.” (Participant D, SGLC 2).

Participants also acknowledged the considerable effort made by lecturers, particularly those with limited prior experience of digital technologies, who adapted to online teaching despite the sudden transition.

One participant reflected:

“And also, to show that they cared neh Mam, our first‐year lecturers were, they are not so young, neh… they were older, but then they pushed themselves to get used to the system, to be there for us, I think that’s a bonus, They were there…” (Participant B, SGLC 2).

These behaviours were interpreted by participants as evidence of lecturers’ commitment, resilience and caring presence during an unprecedented period of disruption.

#### 3.4.2. Subtheme 1.2: Participants Experienced a Lack of Consideration at the Academic Level

Although participants recognised the efforts made by many lecturers, they also described experiences in which they felt academically unsupported during online learning. Several participants perceived that greater emphasis was placed on delivering academic content than on ensuring that students could meaningfully access and engage with the learning materials. They described inconsistent online teaching approaches, limited interaction during virtual sessions and a lack of structured learning platforms, which negatively influenced their learning experiences.

Participants expressed their frustrations in the following manner:

“It could have been good if we were using a different platform but for this, because of the WhatsApp, it was completely terrible. Because you would get the lecturer asking a question at 12 o clock, and by one o clock people are still responding to that same question and we have moved on from that. (Participant C, CHBC 2).

Participants explained that they often relied on peer‐created WhatsApp groups to clarify content and obtain academic information, suggesting that they assumed responsibility for maintaining their own learning during the pandemic. These experiences contributed to perceptions that lecturers’ caring presence was diminished when communication and academic guidance were inconsistent.

#### 3.4.3. Subtheme 1.3: Participants Experienced That Their Mental Well‐Being Was Not Considered During the Pandemic

Beyond academic support, participants expressed a need for greater emotional support during the COVID‐19 pandemic. They believed that the primary institutional focus was on maintaining academic continuity, while the psychological impact of the pandemic on nursing students received limited attention.

One participant explained:

“…it somewhat feels like they did not put like our mental health or emotions into considerations, it was all about getting the job done” (Participant C, CHBC 2).

Participants described feelings of isolation, uncertainty and emotional strain associated with both the pandemic and the transition to online learning. They suggested that greater acknowledgement of students’ emotional well‐being would have strengthened lecturers’ caring presence and contributed to a more supportive learning environment.

Overall, Theme 1 demonstrates that nursing students understood lecturers’ caring presence as extending beyond the delivery of academic content. Caring presence encompassed accessible communication, responsiveness, empathy, emotional support and a commitment to meeting students’ academic and personal needs during a period of significant disruption.

### 3.5. Theme 2: Institutional and Technological Challenges Influenced Students’ Perceptions of Lecturers’ Caring Presence During the Transition to Online Learning

Participants described the rapid transition to online learning during the COVID‐19 pandemic as challenging and, at times, disorganised. They perceived that the NEI was insufficiently prepared for the sudden shift from face‐to‐face to online teaching, which negatively affected their learning experiences and influenced their perceptions of lecturers’ caring presence. Despite these challenges, participants also identified positive aspects of learning from home and reflected on how greater institutional and technological support could have enhanced both their learning experience and lecturers’ ability to demonstrate caring presence.

#### 3.5.1. Subtheme 2.1: The Rapid Transition to Online Learning Exposed Institutional Unpreparedness

Participants described the transition to online learning as abrupt and poorly coordinated. They believed that the nursing college lacked the technological infrastructure, learning management systems and institutional preparedness necessary to support effective online teaching. These challenges affected communication between lecturers and students and influenced participants’ perceptions of lecturers’ ability to provide consistent academic support.

One participant compared their previous higher education institution with the nursing college:

“The experience was quite challenging… I came from another institution where online learning was executed properly. Here the connection was poor, the applications were not user‐friendly, and most of the teaching happened through WhatsApp. There wasn’t one central platform where you could find your modules and activities. Sometimes lecturers struggled to connect to the internet, and some students couldn′t attend because they didn′t have data.” (Participant C, SGLC 2)

Participants recognised that many of these challenges extended beyond individual lecturers and reflected broader institutional limitations. Nevertheless, the lack of coordinated systems influenced their experience of online learning and affected how lecturers’ caring presence was experienced in the virtual environment.

#### 3.5.2. Subtheme 2.2: Learning From Home Provided Flexibility and Greater Independence

Despite the challenges associated with online learning, participants identified several positive experiences related to studying from home. They appreciated the flexibility, reduced travel costs and increased responsibility for managing their own learning. Access to recorded learning materials also enabled participants to revisit lectures at their own pace, promoting independent learning.

One participant reflected:

“Everything that was explained was on my phone. I could go back to it a week later and listen again. That made it much easier to understand the content because I wasn′t just relying on the slides.” (Participant H, CHBC 1)

Another participant valued the flexibility associated with online learning:

“I think the best part was doing the activities at your own pace.” (Participant C, SGLC 2)

Although participants appreciated the autonomy that online learning afforded, they emphasised that flexibility was most beneficial when accompanied by accessible lecturers who remained available to provide guidance and academic support. This reinforced their understanding that caring presence extended beyond the delivery of content to include ongoing engagement with students’ learning needs.

#### 3.5.3. Subtheme 2.3: Limited Technological Resources Disrupted Learning and Reduced Access to Support

Participants consistently identified inadequate technological resources as a major barrier to successful online learning. Many relied on personal mobile phones to attend classes, while limited access to data, devices, reliable internet connectivity and electricity interruptions reduced their ability to participate fully in online learning activities.

One participant explained:

“It would have been better if they gave us monthly data like other institutions. During load shedding there was no network, and by the time the electricity came back, the class was already over.” (Participant C, SGLC 2)

Another participant suggested:

“I wish they supplied data and devices for those who needed them, but data for everyone.” (Participant B, CHBC 1)

Participants believed that earlier provision of devices, internet access and technical support would have eased the transition to online learning and strengthened communication between lecturers and students. They perceived that improved technological support would have enabled lecturers to demonstrate their caring presence more effectively by ensuring that students could consistently access learning opportunities.

Theme 2 illustrates that participants’ perceptions of lecturers’ caring presence were shaped not only by lecturers’ individual behaviours but also by the institutional and technological context in which online learning occurred. While participants recognised lecturers’ efforts to adapt during an unprecedented period, they believed that institutional preparedness, reliable digital infrastructure and equitable access to technological resources were essential to supporting caring, effective and inclusive online nursing education.

### 3.6. Theme 3: Participants Identified Strategies to Strengthen Lecturers’ Caring Presence in Future Online and Blended Learning Environments

Participants proposed several recommendations to improve future online learning within nursing education institutions. Their recommendations extended beyond technological improvements to include stronger institutional leadership, enhanced lecturer and student preparedness, equitable access to digital resources and greater responsiveness to students’ academic and emotional needs. Collectively, these recommendations reflected participants’ vision of a caring learning environment that would be better equipped to support nursing students during future disruptions. These findings align with the Dream, Design and Destiny phases of AI by focussing on envisioning and designing improved educational practices.

#### 3.6.1. Subtheme 3.1: Participants Expected Nursing Education Institutions to Be Prepared to Support Students During Future Emergencies

Participants believed that nursing education institutions should develop comprehensive contingency plans to ensure continuity of teaching and learning during future crises. They expected institutions to learn from the experiences of the COVID‐19 pandemic by establishing appropriate digital infrastructure, academic support systems and student support services before another emergency occurred.

One participant explained:

“We were not the first group. The first group also came during COVID, so they should have used that experience to develop strategies before we arrived. Instead, it felt like they were unprepared.” (Participant C, CHBC 1)

Participants also highlighted that preparedness extended beyond academic planning to include technological support for students with limited digital literacy.

As one participant reflected:

“Before coming to college, I didn′t even have a smartphone. Someone had to help me learn how to use the devices and complete the activities, and that built my confidence.” (Participant A, SGLC 2)

Participants further believed that institutional preparedness required management to provide lecturers and students with adequate technological resources to support effective online learning.

“The problem starts with management because they are not providing the resources and equipment needed for lecturers and students, but they still expect the required outcomes to be achieved.” (Participant F, CHBC 1)

These findings suggest that students perceived institutional preparedness as a prerequisite for lecturers to demonstrate caring presence effectively during periods of educational disruption.

#### 3.6.2. Subtheme 3.2: Participants Recommended Continuous Digital Training for Both Lecturers and Students

Participants consistently emphasised the importance of ongoing training in digital technologies for both lecturers and nursing students. They believed that familiarity with learning management systems and online teaching platforms would improve communication, reduce technical disruptions and enhance the overall learning experience.

One participant stated:

“I feel that lecturers should have been trained on the different platforms used to engage with students, and students should also have been given the opportunity to learn how to use them.” (Participant E, CHBC 1)

Similarly, another participant commented:

“If lecturers had received training on how to use these applications more efficiently, we wouldn′t have spent so much time trying to connect before classes could even begin.” (Participant C, SGLC 2)

Participants viewed digital competence as an essential component of contemporary nursing education and believed that regular training would strengthen lecturers’ ability to maintain caring, supportive and effective learning relationships in online environments.

#### 3.6.3. Subtheme 3.3: Participants Advocated for Responsive Leadership and Collaborative Decision‐Making

Participants expressed a desire for nursing education institutions to adopt more responsive, transparent and student‐centred leadership approaches. They believed that students should be actively consulted regarding educational decisions, particularly during periods of rapid organisational change. Open communication between management, lecturers and students was viewed as fundamental to creating a caring educational environment.

One participant explained:

“Management needs to prepare better for new students in every aspect. They should communicate with us, listen to us, and consider our opinions as students.” (Participant E, SGLC 1)

Participants also encouraged institutional leaders to benchmark against other higher education institutions that had successfully implemented online learning during the pandemic.

As one participant suggested:

“There are other institutions where online learning worked well. I think we should look at what they are doing and learn from them.” (Participant E, CHBC 1)

Participants believed that continuous institutional learning, evidence‐informed decision‐making and meaningful student engagement would strengthen organisational preparedness and promote lecturers’ caring presence within future online learning environments.

Theme 3 demonstrates that participants envisioned caring online nursing education as a shared institutional responsibility rather than solely the responsibility of individual lecturers. They believed that caring presence could be strengthened through proactive institutional planning, investment in digital infrastructure, continuous technological training, responsive leadership and meaningful collaboration between students, lecturers and management. Collectively, these recommendations provide practical guidance for nursing education institutions seeking to develop resilient, inclusive and caring learning environments capable of responding to future educational disruptions.

The findings demonstrate that nursing students perceived lecturers’ caring presence as a multidimensional concept encompassing academic support, emotional responsiveness, effective communication and accessibility. Participants acknowledged lecturers’ commitment during the rapid transition to online learning but highlighted how institutional preparedness, technological resources and organisational leadership influenced the extent to which caring presence could be experienced [74]. The recommendations generated by participants provide practical guidance for strengthening caring, inclusive and resilient online and blended nursing education in future.

## 4. Discussion

This study explored nursing students’ perceptions of lecturers’ caring presence during online learning at two campuses of a public nursing college in Gauteng Province, South Africa, during the COVID‐19 pandemic. The findings demonstrate that caring presence extends beyond the delivery of academic content and encompasses meaningful interpersonal relationships characterised by accessibility, responsiveness, empathy, emotional support and effective communication. Importantly, participants’ experiences also highlighted that lecturers’ caring presence was shaped not only by individual lecturer behaviours but also by institutional preparedness, technological infrastructure and organisational support during the rapid transition to online learning.

The central finding of this study was that nursing students experienced both caring and uncaring interactions with their lecturers during online learning. Participants associated caring presence with lecturers who remained accessible, communicated regularly, responded promptly to questions and emails and demonstrated genuine concern for students’ academic progress and personal well‐being. These relatively simple interpersonal behaviours helped students feel recognised, valued and supported despite the physical separation imposed by online learning.

These findings are consistent with previous research demonstrating that meaningful lecturer–student relationships remain fundamental to successful online learning [75, 76]. Although technology provides opportunities for collaborative and flexible learning, the quality of interpersonal engagement continues to influence students’ learning experiences and perceptions of support [77]; [78]. During the COVID‐19 pandemic, nursing education institutions rapidly redesigned teaching to maintain educational continuity, requiring lecturers to adopt alternative ways of expressing care through virtual communication, online consultations and regular electronic contact [79, 80]. The present study extends these findings by demonstrating that nursing students interpreted lecturers’ responsiveness, availability and consistent communication as tangible expressions of caring presence within the online environment.

However, participants also described experiences in which caring presence appeared diminished. While academic activities continued throughout the pandemic, many students perceived that their emotional well‐being received limited attention. Participants reported feeling isolated, overwhelmed and emotionally unsupported during a period characterised by considerable uncertainty and disruption. These findings suggest that caring presence in online nursing education should encompass emotional support alongside academic facilitation.

This finding is supported by studies demonstrating that the COVID‐19 pandemic increased anxiety, uncertainty and psychological distress among nursing students, with negative consequences for academic performance and overall well‐being [81, 82]. Similarly, Wingfield et al. [83] reported that students from South Africa, Canada and Sweden experienced feelings of isolation during online learning because opportunities for informal interactions with peers and lecturers were substantially reduced. As nursing education has traditionally relied on close interpersonal engagement, the sudden absence of face‐to‐face interaction challenged both teaching practices and students’ sense of connectedness. The present findings reinforce the importance of intentionally integrating emotional support and relational teaching strategies into online and blended nursing education.

The findings further suggest that caring presence should not be viewed solely as an individual characteristic of lecturers but rather as a shared institutional responsibility. Participants believed that inadequate academic support, limited access to technological resources and insufficient institutional preparedness reduced lecturers’ ability to provide effective support during the transition to online learning. Similarly, Darch et al. [84] emphasised that nursing education institutions have a responsibility to provide academic, psychological and practical support that promotes students’ well‐being throughout their educational journey. Consistent with Sineke et al. [85] and Sitzman and Leners [86], the findings indicate that supportive educational environments foster students’ experiences of being cared for, which may subsequently influence the caring relationships they develop within professional nursing practice.

Participants also reflected on the rapid transition from traditional classroom teaching to online learning, acknowledging both the challenges and unexpected benefits of this educational shift. While the transition was widely perceived as disorganised because of technological limitations and institutional unpreparedness, students appreciated the flexibility, independence and convenience associated with learning from home. These findings mirror those reported by Thapa et al. [11] and Harerimana and Mtshali [87] who found that the sudden implementation of online learning posed considerable challenges for both students and educators in contexts where digital education had not previously been well established. Similar experiences within the South African nursing education context suggest that successful implementation of online and blended learning requires careful planning, adequate infrastructure and ongoing institutional support.

Participants further emphasised that equitable access to technology was essential for meaningful participation in online learning. Limited access to internet connectivity, digital devices, technical support and digital literacy training constrained learning opportunities and contributed to unequal educational experiences. These findings support those of Chipps et al. [88] and Bdair [89], who reported that limited digital competence and uncertainty regarding technology remain challenges within nursing education, particularly among more experienced educators. Likewise, Brown et al. [90] and Nguyen and Duong [91] argued that although nursing education institutions increasingly recognise the importance of developing digital competence, implementation remains inconsistent internationally. The present study extends this evidence by highlighting that technological preparedness not only facilitates online learning but also enables lecturers to demonstrate caring presence through consistent communication, accessibility and meaningful engagement with students [92].

### 4.1. Strengths

This study provides an in‐depth understanding of nursing students’ perceptions of lecturers’ caring presence during online learning within the South African nursing education context. The qualitative, contextual design enabled participants to share rich descriptions of their lived experiences, providing nuanced insights into the academic, emotional and institutional factors that shaped their perceptions. The use of focus group interviews encouraged interaction among participants, allowing shared experiences and diverse perspectives to emerge.

A further strength of the study was the use of AI to guide data collection. This approach encouraged participants to reflect not only on the challenges they experienced during online learning but also on positive experiences and opportunities for improving future nursing education. Consequently, the study generated practical, participant‐informed recommendations for strengthening caring presence in online and blended learning environments.

The inclusion of nursing students from two campuses enhanced the diversity of perspectives captured and strengthened the transferability of the findings to similar nursing education contexts. Collectively, these strengths contribute to the growing body of evidence informing the design of caring, student‐centred and digitally enabled nursing education.

### 4.2. Limitations

This study has several limitations that should be considered when interpreting the findings. First, participants were recruited from only two campuses of the Gauteng College of Nursing, which may limit the transferability of the findings to other nursing education institutions or geographical contexts. Second, the study focused specifically on the experiences of third‐year nursing students who had participated in online learning during the COVID‐19 pandemic.

Consequently, the findings reflect perceptions of emergency remote teaching during an unprecedented period and may not fully represent experiences of online or blended learning implemented under routine educational conditions.

Despite these limitations, the study provides valuable insights into nursing students’ perceptions of lecturers’ caring presence and offers practical recommendations for strengthening caring, inclusive and resilient online and blended nursing education.

### 4.3. Recommendations

The findings of this study provide practical recommendations for strengthening lecturers’ caring presence in online and blended nursing education. These recommendations are directed towards nursing education institutions, educators, curriculum developers, healthcare organisations and researchers to support the development of caring, equitable and technologically enabled learning environments.

### 4.4. Recommendations for Nursing Education and Practice

NEIs should adopt a proactive approach to integrating caring pedagogy within online and blended learning environments. Continuous professional development should be provided to lecturers to strengthen their digital competence, confidence in using learning management systems and ability to demonstrate caring presence through effective online communication, timely feedback and student engagement.

Nursing students should receive ongoing academic, technological and psychosocial support throughout their programme to facilitate successful learning and promote resilience. Early identification of students requiring additional academic or technological assistance should form part of routine student support services.

Institutions should also ensure equitable access to digital devices, internet connectivity and technical support from the commencement of nursing training to minimise barriers to participation in online learning.

### 4.5. Recommendations for Curriculum Development

The findings highlight the need to strengthen digital competence within undergraduate nursing education. A foundational ICT module should be incorporated into the Diploma in Nursing curriculum during the first year of study and reinforced throughout the programme. This would assist students in developing essential digital literacy skills required for both academic learning and contemporary clinical practice.

The curriculum should also intentionally integrate caring, compassion, resilience and therapeutic communication within online and blended learning approaches to ensure that caring values remain central to nursing education irrespective of the teaching modality.

### 4.6. Recommendations for Institutional Leadership and Policy

NEIs should develop comprehensive institutional strategies that support the delivery of caring online and blended education during both routine teaching and future emergencies. Institutional preparedness should include investment in digital infrastructure, learning management systems, student support services, and contingency planning.

College leadership should strengthen communication between management, lecturers and students by promoting transparent decision‐making and actively involving students in educational planning. Benchmarking against other nursing education institutions with established digital learning systems may further support continuous quality improvement.

At a national level, nursing education policies should incorporate standards for digital readiness, online teaching competencies and strategies that support caring pedagogy within technology‐enhanced learning environments.

### 4.7. Recommendations for Nursing Education Research

Further qualitative and quantitative research should explore lecturers’ caring presence across diverse public and private nursing education institutions to determine whether similar experiences exist in different educational contexts.

Longitudinal studies examining students’ experiences of blended learning beyond the COVID‐19 pandemic would provide valuable insights into the sustainability of caring presence in evolving educational environments. Future intervention studies evaluating strategies aimed at strengthening lecturers’ caring presence, digital competence and institutional preparedness are also recommended.

## 5. Conclusion

The COVID‐19 pandemic fundamentally transformed nursing education and highlighted the importance of sustaining caring relationships within technology‐enhanced learning environments. This study demonstrated that nursing students perceived lecturers’ caring presence as extending beyond academic instruction to include accessibility, responsiveness, empathy, emotional support and meaningful communication. While participants acknowledged lecturers’ commitment during the rapid transition to online learning, they also recognised that caring presence was influenced by broader institutional factors, including organisational preparedness, digital infrastructure, leadership and equitable access to technological resources.

The findings emphasise that caring presence in nursing education is both relational and structural. It is expressed through lecturers’ interpersonal interactions with students but is simultaneously enabled by institutional systems that support effective teaching, learning and student well‐being. Strengthening digital competence, investing in technological infrastructure, embedding caring pedagogy within online and blended learning and providing comprehensive academic and psychosocial support are, therefore, essential for preparing resilient nursing education systems capable of responding to future disruptions.

This study contributes to the growing international body of literature on caring in nursing education by providing evidence from a South African public nursing college context, a setting that remains underrepresented in the literature. The findings offer practical guidance for nursing education institutions, curriculum developers and policymakers seeking to strengthen caring, inclusive and technology‐enhanced learning environments while preserving the relational values that underpin the nursing profession.

## Author Contributions

Thembile Precious Ndlovu: conceptualisation, methodology, validation, formal analysis, investigation, resources, data curation, writing–original draft, writing–review and editing, visualization, and project administration. Charlene Downing: conceptualisation, methodology, validation, formal analysis, supervision, and writing–review and editing.

## Funding

This research did not receive any specific grant from funding agencies in the public, commercial or not‐for‐profit sectors.

The authors declare that no financial support was received for this article’s research, authorship or publication.

## Disclosure

This article was previously presented as a thesis in ProQuest.

## Ethics Statement

The study received approval from the Faculty of Health Sciences Research Ethics Committee (REC‐1670‐2022) and the Higher Degrees Committee (HDC‐01‐55‐2022) of the University of Johannesburg to commence. The researcher then obtained permission from the Gauteng Department of Health Research and Provincial Protocol Review Committee (PPRC), and permission was granted (GP202208_072).

## Conflicts of Interest

The authors declare no conflicts of interest.

## Data Availability

The data that support the findings of this study are available on request from the corresponding author. The data are not publicly available due to privacy or ethical restrictions.
